# Trivial and nontrivial error sources account for misidentification of protein partners in mutual information approaches

**DOI:** 10.1038/s41598-021-86455-0

**Published:** 2021-03-25

**Authors:** Camila Pontes, Miguel Andrade, José Fiorote, Werner Treptow

**Affiliations:** Laboratório de Biologia Teórica e Computacional (LBTC), Universidade de Brasília DF, Brasília, Brazil

**Keywords:** Computational biophysics, Protein analysis, Statistical methods

## Abstract

The problem of finding the correct set of partners for a given pair of interacting protein families based on multi-sequence alignments (MSAs) has received great attention over the years. Recently, the native contacts of two interacting proteins were shown to store the strongest mutual information (MI) signal to discriminate MSA concatenations with the largest fraction of correct pairings. Although that signal might be of practical relevance in the search for an effective heuristic to solve the problem, the number of MSA concatenations with near-native MI is large, imposing severe limitations. Here, a Genetic Algorithm that explores possible MSA concatenations according to a MI maximization criteria is shown to find degenerate solutions with two error sources, arising from mismatches among (i) similar and (ii) non-similar sequences. If mistakes made among similar sequences are disregarded, type-(i) solutions are found to resolve correct pairings at best true positive (TP) rates of 70%—far above the very same estimates in type-(ii) solutions. A machine learning classification algorithm helps to show further that differences between optimized solutions based on TP rates are not artificial and may have biological meaning associated with the three-dimensional distribution of the MI signal. Type-(i) solutions may therefore correspond to reliable results for predictive purposes, found here to be more likely obtained via MI maximization across protein systems having a minimum critical number of amino acid contacts on their interaction surfaces (N > 200).

## Introduction

**Figure 1:**
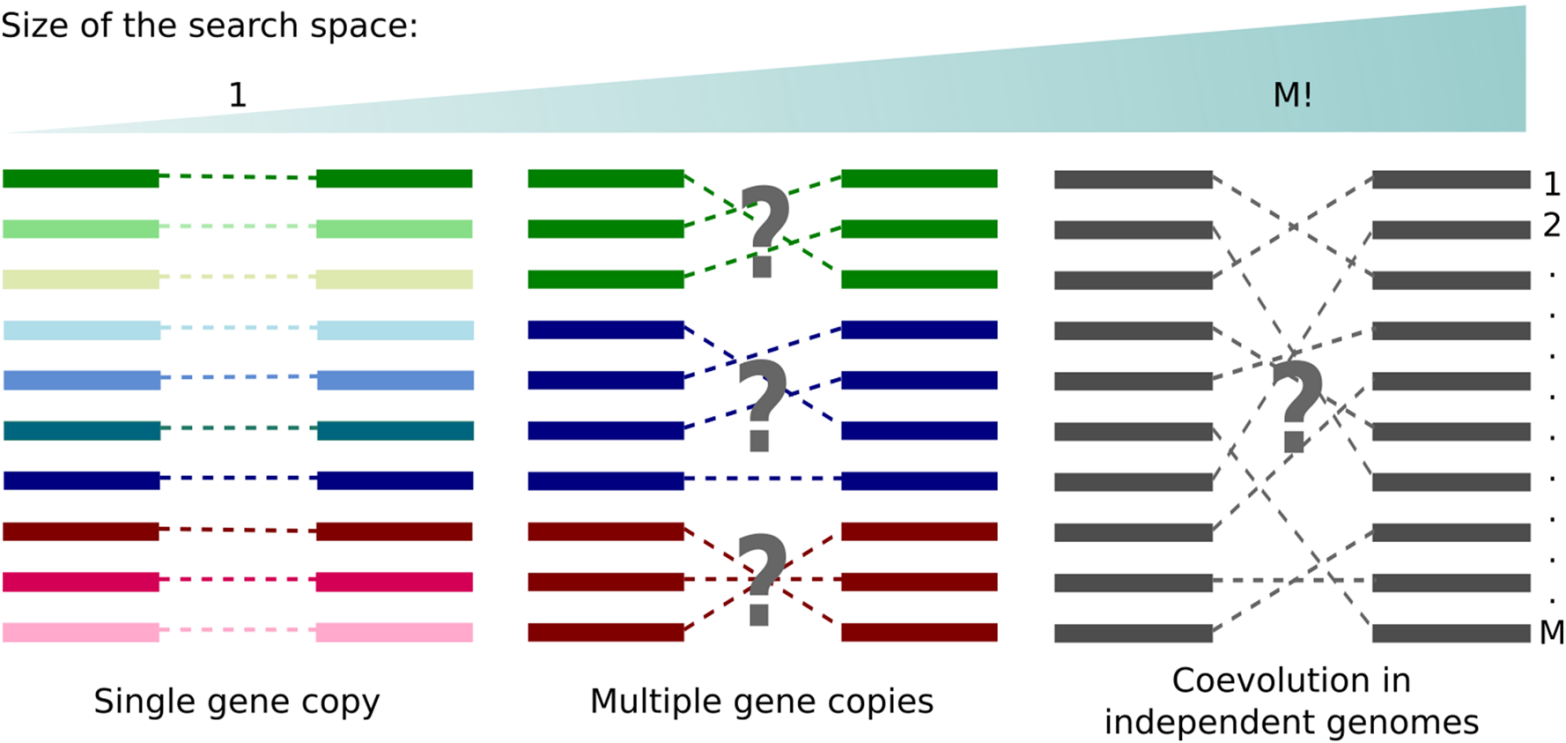
Different scenarios for protein partners determination from multi-sequence alignments. The correct set of partners is known for systems with a single gene copy per genome and unknown for systems involving multiple (paralogous) sequences within the same genome or multiple sequences across independent genomes. This figure was created with Inkscape (https://inkscape.org/).

Coevolution of proteins A and B translates itself into a series of homologous primary-sequence variants encoding coordinated compensatory mutations and, therefore, a specific set of protein–protein interactions between members of family A and members of family B. The problem of resolving specific protein partners based on multi-sequence alignments (MSAs) has received great attention over the years^1,2^. Ingenious approaches based on the correlation of phylogenetic trees^3–5^ and profiles^6^, gene colocalization^7^ and fusions^8^, maximum coevolutionary interdependencies^9^ and correlated mutations^10,11^, maximization of the interfamily coevolutionary signal^12^, iterative paralog matching based on sequence energies^13^ and expectation–maximization^14^ have been developed and applied to resolve interaction partners in single or multiple (paralogous) gene copies in the same genome. Despite these advances, the problem of protein partners prediction remains unsolved for large sequence ensembles in general, especially for the case of protein coevolution across independent genomes—examples are phage proteins and bacterial receptors, pathogen and host-cell proteins, neurotoxins and ion channels, to mention a few. The problem lacks any suitable solution especially because an effective heuristic to search for the correct set of protein partners across the space of M! potential matches still misses in case of large number of sequences M (Fig. 1).

In a previous investigation, we showed that the coevolutive information encoded on the interacting amino acids of proteins A and B can be useful to discriminate the correct set of protein partners based on MSAs, in contrast to other evolutive and stochastic sources spread over their sequences^15^. When compared to other sources, the coevolutive information is the strongest signal to distinguish protein partners derived from coevolution within the same genome and, likely, the unique indication available in the case of protein interactions in independent genomes. We showed that physically-coupled amino acids at the molecular interface of A and B store the largest per-contact mutual information ($\hat{I}_{AB}$) to discriminate MSA concatenations with the largest expectation fraction of correct interaction partners—a result that was found to hold for various definitions of intermolecular contacts and binding modes. Although that information content might be of practical relevance in the search of an effective heuristic to resolve specific protein partners, the degeneracy $\omega$, i.e., the number of MSA concatenations with a similar amount of ${\widehat{I}}_{AB}$ to the native concatenation is expected to be large ($\omega \gg M$), imposing severe limitations to that purpose.

Here, we investigate that hypothesis accordingly for a variety of protein families, including obligate and non-obligate complexes. It is worth emphasizing that the aim of this work is not to provide a method for the prediction of protein–protein interactions nor protein–protein interfaces, hence it differs from the studies in which sequence covariance is used to predict three-dimensional amino acid contacts or to infer specific interactions for a set of paralogs. Instead, we want to qualitatively explore the MI degeneracy in the space of possible protein partners associations between two interacting protein families. To approach that, we analyze a set of converged trajectories produced by a Genetic Algorithm (GA) that maximizes ${\widehat{I}}_{AB}$ starting from scrambled MSA concatenations of protein families with known partners in the same genome. Consistent with the expected degeneracy of ${\widehat{I}}_{AB}$, GA optimizations show two subspaces of MSA concatenation solutions: subspace (i), which consists of optimized solutions with a trivial error source arising from mismatches among similar sequences; and subspace (ii), which consists of optimized solutions with a non-trivial error source due to mismatches among non-similar sequences. By disregarding mistakes made among similar sequences, protein partners are resolved at best true-positive (TP) rates of ~ 70% in type-(i) optimizations – far above best TP rates in type-(ii). Type-(i) and -(ii) solutions are found to be functionally distinct from each other, with the former presenting a larger near-native content of mutual information correctly distributed among amino acid contacts. Particularly important, that finding supports the notion that differences between optimized solutions based on TP rates have a biological meaning associated with the amount of functional information and its spatial distribution. Type-(i) solutions may therefore correspond to reliable results for predictive purposes^1^, more likely obtained via ${\widehat{I}}_{AB}$ maximization across protein systems found here to have a minimum critical number of amino acid contacts on their interaction surfaces (N > 200).

## Results and discussion

**Figure 2:**
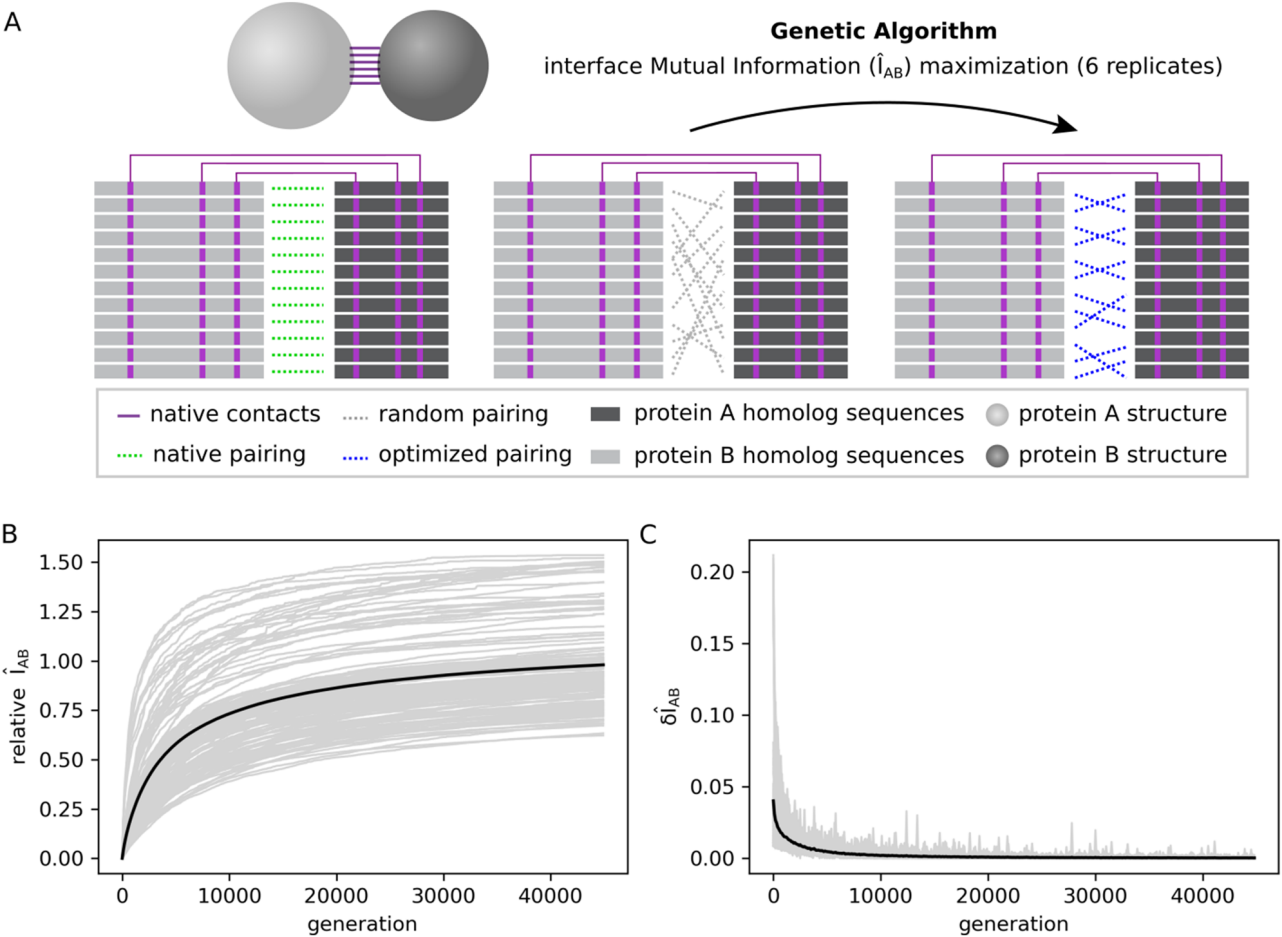
Interface mutual information ($\hat{I}_{AB}$) optimization trajectories. (**A**) Scheme showing $\hat{I}_{AB}$ optimization process starting from a scrambled multi-sequence alignment (MSA) concatenation (in gray) and reaching an optimized concatenation (in blue). Only physically coupled MSA position pairs (shown in purple) are taken into account. (**B**) Optimization trajectories for 26 protein systems. For each system, there are six trajectories with different starting points. The $\hat{I}_{AB}$ normalized by the native interface mutual information (relative $\hat{I}_{AB}$) is plotted against the number of generations of the genetic algorithm (gray lines). The average trajectory over all complexes is shown in black. (**C**) First-order derivative of the optimization trajectories shown in (**B**). The derivatives of individual trajectories are shown in gray, while the average derivative over all trajectories is shown in black. This figure was generated with Inkscape (https://inkscape.org/) and matplotlib v3.1.2 (https://matplotlib.org/).

In search of an effective heuristic to resolve specific protein partners based on MSAs with large numbers of sequences, the degeneracy of the per-contact mutual information $\hat{I}_{AB}$ was investigated here across 26 independent protein families with known interaction partners in the same genome (see “Methods” and Table S1). To approach that, we have performed optimization trajectories produced by a Genetic Algorithm (GA, see “Methods” and Algorithm S1) that starts from a random concatenation of MSA A and MSA B, and maximizes $\hat{I}_{AB}$ by performing small changes in the MSA concatenation iteratively (Fig. 2A). Accordingly, Fig. 2B shows 156 optimization trajectories with convergence obtained after 45,000 generations as indicated by their average time derivative $\delta \hat{I}_{AB} \le 0.001$ in Fig. 2C. The average trajectory converges at ~ 98% of the $\hat{I}_{AB}$ reference value in the native concatenation z^*^.

**Figure 3:**
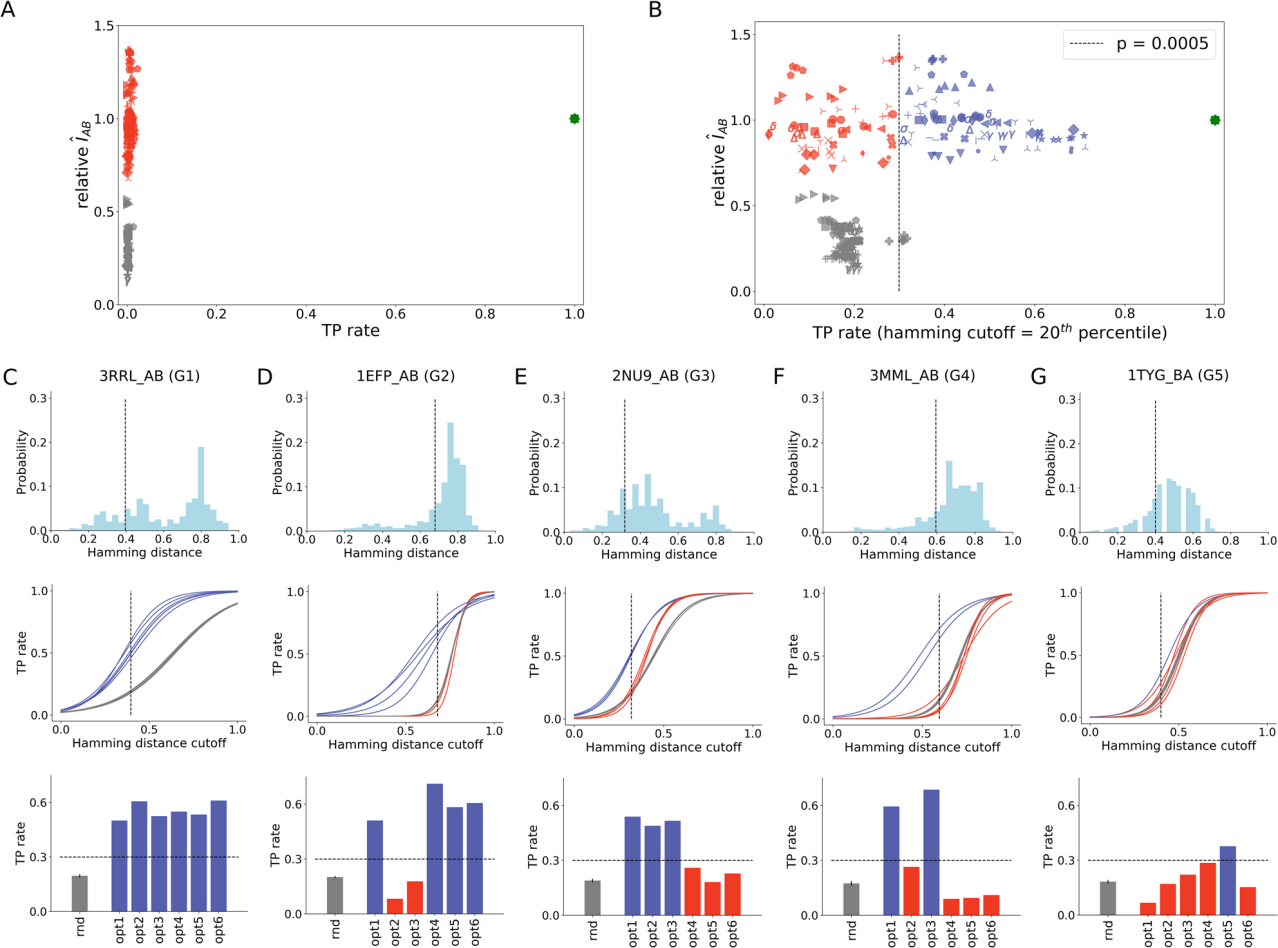
Evaluation of optimized MSA concatenations. (**A**) True positive (TP) rate of random, optimized and native MSA concatenations. (**B**) Reassessed TP rate of random, optimized and native MSA concatenations by discounting wrong pairings among sequences with Hamming distance within the 20th percentile of the distance distribution. Optimized solutions with TP rate greater than 30% (p = 0.0005) are shown in blue, while optimized solutions with TP rate lower than 30% are shown in red. Random solutions are shown in gray. (**C**–**G**) Hamming distance distribution of MSA B, TP rates versus Hamming distance discounts (the 20th percentile is shown with a dashed line), and TP rates of random (rnd) and optimized (opt1–6) solutions for the 20th percentile Hamming distance cutoff shown for representative systems: 3RRL_AB (**C**), 1EFP_AB (**D**), 2NU9_AB (**E**), 3MML_AB (**F**), and 1TYG_BA (**G**). This figure was generated using matplotlib v3.1.2 (https://matplotlib.org/ ).

Despite presenting near-native values of $\hat{I}_{AB}$, optimized solutions fail at pairing sequences correctly in consequence of the degeneracy of the space of possible MSA models constrained by the $\hat{I}_{AB}$ maximization criteria. As made clear in Fig. 3A, there are three groups of solutions: one group of scrambled concatenations with 0% TP rate and low values of $\hat{I}_{AB}$ (in gray), one group of optimized concatenations with 0% TP rate and near-native $\hat{I}_{AB}$ (in red), and one group of native concatenations with 100% TP rate and native $\hat{I}_{AB}$ (in green). Careful inspection of the data reveals that the presence of similar sequences in MSA B contributes to that high error rate by yielding similar optimized values of $\hat{I}_{AB}$ when paired with a given sequence in MSA A. Indeed, reassessment of TP rates by disregarding mistakes made among sequences at the 20th percentile of Hamming distances distribution (see “Methods”—Fig. 9) allows regrouping of solutions into a subspace (i) with TP rates larger than 30% (Fig. 3B). As a measure of correlation, it is not surprising that mutual information is degenerate given that trivial source of error. Unexpected however is the fact that degeneracy may also involve another subspace of optimized solutions (ii) related to the non-trivial mismatch of sequences at larger Hamming distances. Supporting that notion, protein partners prediction at better TP rates (> 30%) demands a larger fraction of sequence mismatches (above the 20th percentile) to be discounted in optimized solutions (ii). As shown in Supporting Information, conclusions about subspaces (i) and (ii) hold for mismatches definitions using other Hamming distance cutoffs (Figure S1).

**Figure 4:**
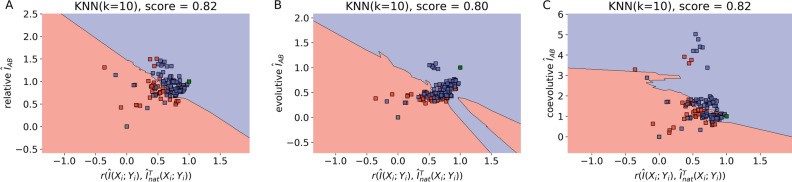
(**A**) Optimized concatenation solutions scattered across the space of relative interface mutual information (MI), $\hat{I}_{AB}$, against Pearson correlation between optimized and native MI vectors, $r(\hat{I}(X_{i} ;\;Y_{i} ),\;\hat{I}_{nat}^{T} (X_{i} ;\;Y_{i} ))$. Type-(i) solutions are shown in red and type-(ii) solutions are shown in blue. The bidimensional space was separated by a k-nearest neighbors (KNN) classification algorithm^16^ (default Python 3 scikit-learn implementation, k = 10, for other k values see Figure S2). Native and scrambled concatenations were plotted afterwards in the same space and are shown in green and gray, respectively. Analogous plots were generated for the evolutive (**B**) and coevolutive (**C**) components of $\hat{I}_{AB}$. The decomposition was performed according to^15^. This figure was generated using sci-kit learn v0.22.2 (https://scikit-learn.org) and mlxtend v0.18.0 (http://rasbt.github.io/mlxtend/).

To get further insights on the mismatch problem reported in Fig. 3, the functional distinction of solutions type-(i) and (ii) was then analyzed according to the three-dimensional distribution of evolutive and coevolutive sources of the mutual information signal. Implicit in the analysis is the assumption that type-(i) solutions must necessarily have a near-native content of mutual information correctly distributed among amino acid contacts i.e., a near-native information content with a high correlation $r(\hat{I}(X_{i} ;\;Y_{i} ), \;\hat{I}_{nat}^{T} (X_{i} ;\;Y_{i} ))$ between the optimized solution vector $\hat{I}(X_{i} ;\;Y_{i} )$ and its native conjugate $\hat{I}_{nat}^{T} (X_{i} ;\;Y_{i} )$. Consistent with that assumption, Fig. 4 shows that the k-nearest neighbor (KNN) machine learning algorithm^16^ discriminates type-(i) and -(ii) solutions with high accuracy ~ 82%, according to their nativelikeness across the space $\hat{I}_{AB} \times r$. A further decomposition analysis reveals the information recovered from type-(i) solutions has larger contents of the evolutive (phylogenetic) and coevolutive signals encoded on the native interacting amino acids of proteins A and B^15^—as also indicated by the high accuracy ~ 82% in which such solutions are effectively classified by the KNN algorithm applied on the correlation space redefined in terms of the specific signals. Here, what is meant by coevolutive signal, as explained in^15^, is the surplus of MI stored in residue pairs at the interface (on average) when compared to the MI stored in residue pairs in general (on average), which is the evolutive, or phylogenetic, signal. For all cases, differentiation is far above the non-significant value of 50% thus supporting the conclusion that differences between optimized solutions based on TP rates may have a biological meaning associated with the amount of functional information recovered and its spatial distribution.

**Figure 5:**
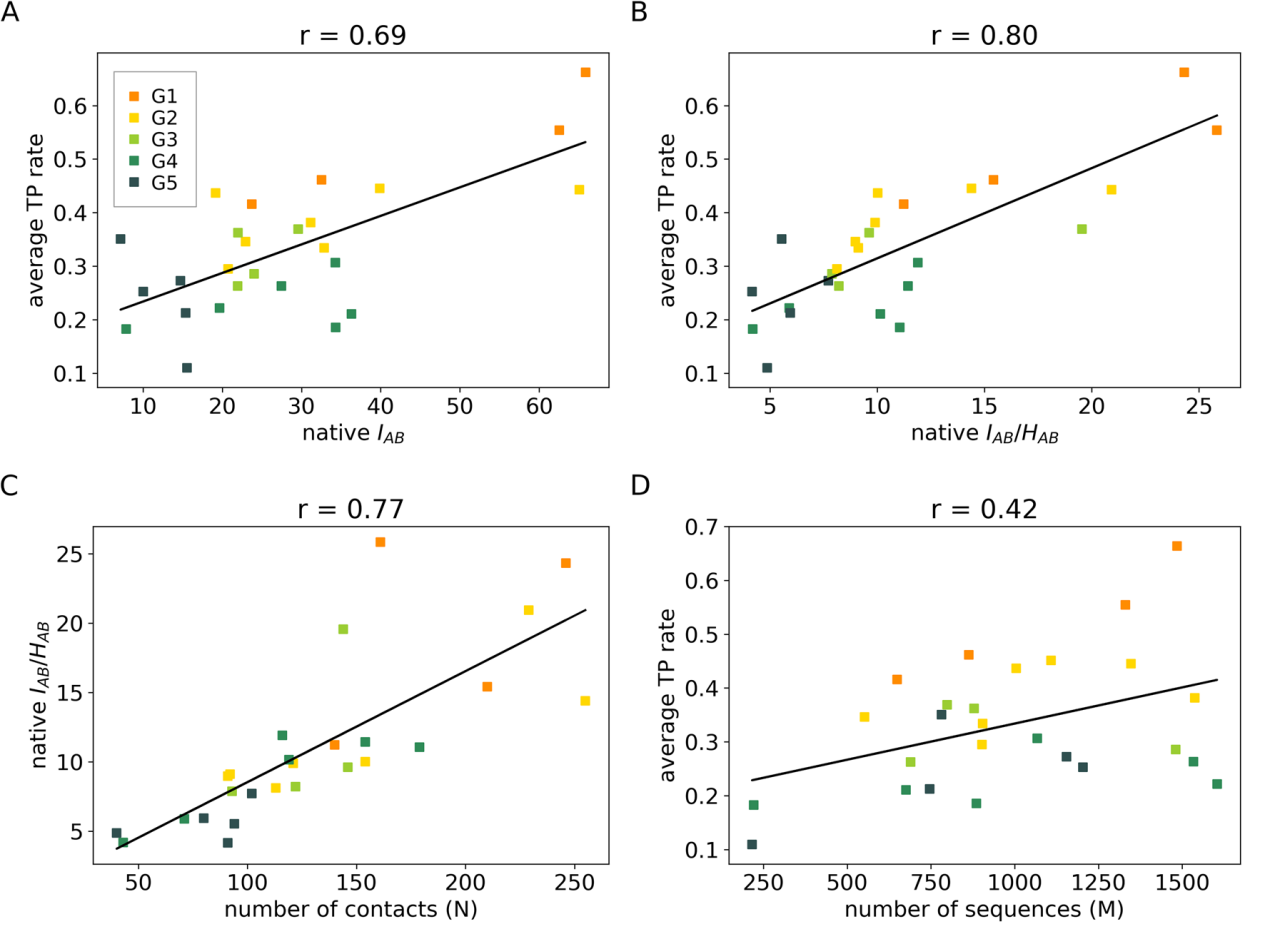
(**A**) Correlation between the true positive (TP) rate of optimized solutions and mutual information (MI) on the interface $I_{AB}$. (**B**) Correlation between TP rate of optimized solutions and $I_{AB}$ regularized by the joint entropy on the interface, $I_{AB} {/}H_{AB}$. (**C**) Correlation between native $I_{AB} /H_{AB}$ and the number of contacts on the interface (N). (**D**) Correlation between TP rate and number of sequences in the alignment (M). Values on the x-axis in A-B were calculated considering the native pairing. TP rates are shown as averages (n = 6) for each system. Systems were colored based on groups G1–5: group 1 is composed by systems with only type-(i) solutions (Fig. 3C and Fig. S3), group 2 by systems with a majority of type-(i) solutions (Fig. 3D and Fig. S4), group 3 by systems with the same proportions of type-(i) and type-(ii) solutions (Fig. 3E and Fig. S5), group 4 by systems with a majority of type-(ii) solutions (Fig. 3F and Fig. S6), and group 5 by systems in which optimized concatenations did not differentiate from the scrambled ones (Fig. 3G and Fig. S7). This figure was generated using matplotlib v3.1.2 (https://matplotlib.org/).

Given the importance that native-like solutions may have in predictive purposes, the propensity of protein systems to produce such optimized solutions was further analyzed according to the content of non-trivial errors. As shown in Fig. 5A,B, protein systems were found to cluster into five distinct groups with average TP rates that strongly correlate with the amount of mutual information at the interaction surface of proteins, with or without regularization by the local joint entropy $H_{AB}$ (see “Methods”). According to that analysis, lower contents of mutual information appear to account for the higher propensity of the system in producing type-(ii) solutions. Because the mutual information content is proportional to the number of amino acid contacts at the protein surface, N (Fig. 5C), this result appears to be consistent with the statistical expectation that the distribution of MI values is broader over systems with fewer degrees of freedom (contacts). More importantly, it indicates N as an important parameter to discriminate suitable protein systems for which maximization of $\hat{I}_{AB}$ may likely produce near-native type-(i) solutions with biological meaning as reported in Fig. 4. The relevance of that parameter becomes clear by noting that the number of MSA sequences (M) does not explain well the content of non-trivial errors across protein clusters (Fig. 5D), despite the well-documented fact that M may significantly impact the accuracy of coevolutionary approaches^17^. The condition N > 200 thus emerges here as one plausible threshold criteria for the classification of protein systems that are suitable for maximization of $\hat{I}_{AB}$ and resolution of protein partners via type-(i) solutions.

So far, our results were obtained from a set of protein families involving unique sequence pairs per genome that may not have coevolved under strong selective pressures towards specificity. To better understand any implicit dependence of the results with that experimental condition, error sources (i) and (ii) were then further investigated in the context of the bacterial two-component system HK-RR featuring highly specific protein–protein interactions across multiple protein copies per genome. More specifically, histidine kinase (HK) and their respective response regulator (RR) are paralogous gene families^13,18,19^, each consisting of multiple sequences sharing significant homology at the primary and tertiary levels. Despite that signature, HK-RR pairs are highly specific within the same genome in consequence of evolutive pressures avoiding crosstalk between independent two-component pathways^20^—as shown by Rowland and Deeds, the evolution of new HK-RR pairs follows rapid sequence divergence immediately after duplication events^21^.

**Figure 6:**
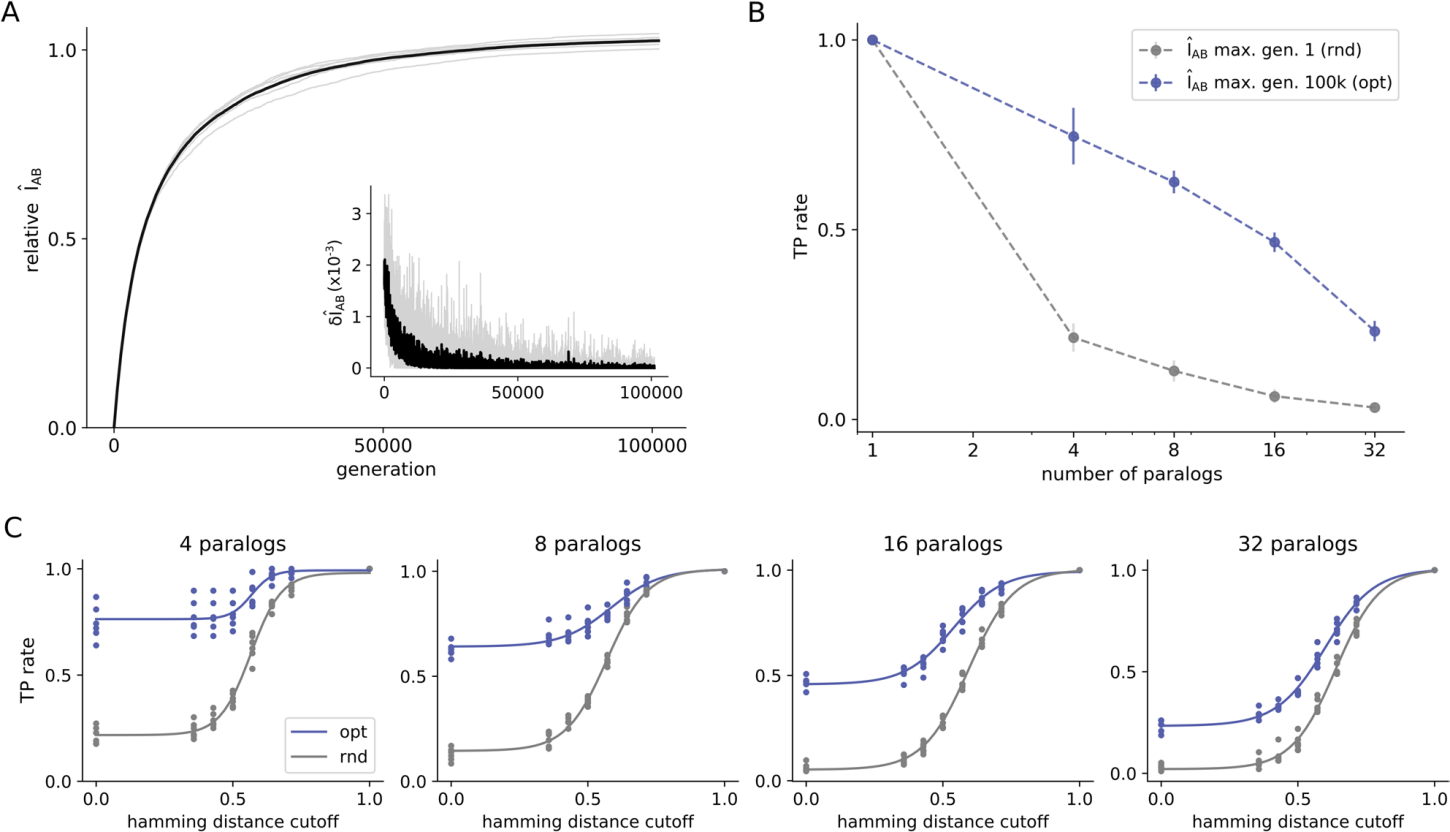
Evaluation of optimized MSA concatenations of the HK-RR paralogs dataset. (**A**) Optimization trajectories for the HK-RR standard dataset. The interface mutual information normalized by the native interface mutual information (relative $\hat{I}_{AB}$) is plotted against the number of generations for optimizations (with 6 replicates each) starting from a solution with a scrambled concatenation within each species. The first derivative of the trajectory is shown in the smaller plot. (**B**) True positive (TP) rate of start (in gray) and final (in blue) solutions after ~ 100,000 rounds of $\hat{I}_{AB}$ maximization. The TP rate is shown in average for bacterial species containing different numbers of paralogs. (**C**) TP rate after disregarding mismatches among sequences considering different Hamming distance cutoffs for bacterial genomes with different numbers of paralogs in the standard HK-RR dataset. The TP rate is shown for both random (rnd) and optimized (opt) MSA concatenations. This figure was generated using matplotlib v3.1.2 (https://matplotlib.org/).

Accordingly, Fig. 6 presents another series of $\hat{I}_{AB}$ optimizations performed on the HK-RR dataset containing around 5000 sequences, coming from ~ 450 bacterial genomes from the P2CS database^22–24^. Optimizations were performed with 6 replicates each, starting from a paired alignment with a randomized pairing within each species. All species were optimized together, which means that each optimization step benefits from the cumulative changes that happened in previous steps (see “Methods”—Fig. 8). As shown in Fig. 6A, optimization to near-native values of $\hat{I}_{AB}$ is attained after ~ 100,000 generations, with $\delta \hat{I}_{AB} < 0.001$.

When analyzing the TP rate for species with different numbers of paralogs, optimized MSA solutions present an improvement over the initial concatenations (Fig. 6B). In this case, TP rates are not null because the degeneracy of (M $\le \hspace{0.17em}$32) paired sequences of paralogs is expected to be significantly smaller than that of (M > 200) paired sequences in Fig. 3. It is interesting to notice that TP rates obtained here by optimizing only the interface MI are only slightly inferior to the same estimates obtained considering full protein MI found in the literature^18^, especially for genomes with a higher number of paralogs. Figure 6C shows further the TP rate of optimized and random MSA concatenations, considering a 20th percentile Hamming distance discount cutoff, for bacterial genomes with different numbers of paralogs. It is possible to observe that random and optimized curves approximate with increasing numbers of paralogs. Extrapolating for cases with more than 32 paralogs, the two curves tend to overlap similarly to what occurs in protein systems in which optimized concatenations did not differentiate from the scrambled ones (Fig. 3G and Fig. S7) and therefore, suggesting that type (i) errors do not contribute to $\hat{I}_{AB}$ degeneracy in HK-RR system. We hypothesize that the lack of type-(i) error originated from mismatches among similar sequences is due to the high specificity of this system.

Results in Fig. 6 appear to rationalize the sharp deterioration of TP rates with the number of sequences in recent investigations of paralogous systems^12–14,18,19^, by hypothesizing it is due to the lack of type-(i) mismatches and the great degeneracy involved. In previous works, Bitbol and coworkers developed an iterative pairing algorithm (IPA) capable of inferring protein partners using either direct coupling analysis (DCA-IPA)^13^, mutual information (MI-IPA)^18^, or phylogeny (Mirrortree-IPA)^19^. When benchmarked for paralog matching on the standard HK-RR dataset, DCA-IPA was as accurate as MI-IPA, and Mirrortree-IPA was even more accurate. The performance of these algorithms, however, drops considerably for species with more than 32 paralogs. The tendency is that the TP rate also drops to zero in a hypothetical genome with hundreds of paralogs^19^, a situation analogous to the results in Fig. 6. In conclusion, results presented in Fig. 6 suggest that paralog matching is only possible because there is usually a small number of paralogous sequences per genome. When extended to genomes with more paralogs, this problem tends to present only type-(ii) solutions, leaving virtually no room for improvement of TP rates.

## Conclusions and future work

Here, we investigate the hypothesis that the coevolutive information encoded on the interacting amino acids of proteins A and B ($\hat{I}_{AB}$) can be useful to discriminate protein partners based on large multi-sequence alignments (MSAs). When compared to evolutive and stochastic sources, $\hat{I}_{AB}$ was previously found as the strongest signal to distinguish protein partners derived from coevolution within the same genome and likely the unique indication in the case of independent genomes^15^. In contrast to other coevolutionary signals that may also be considered in purpose^9,10,12–14^, $\hat{I}_{AB}$ thus corresponds to a small and still important fraction of the total information available in protein sequences making it especially suitable for specific partners inference via fast algorithmic routines. Despite these aspects, the degeneracy of $\hat{I}_{AB}$ is expected to be large and may impose severe limitations to practical applications.

Indeed, $\hat{I}_{AB}$ optimization across the space of possible MSA concatenations is shown here to resolve specific protein partners at very low true positive (TP) rates in consequence of error sources (i) and (ii). As a measure of correlation, it is not surprising that $\hat{I}_{AB}$ is degenerate given trivial mismatches (i) among similar sequences. Unexpected however is the fact that degeneracy may also involve another subspace of optimized solutions (ii) with the non-trivial mismatch of sequences at larger Hamming distances. If trivial error sources are disregarded, further analysis indicates, however, that protein partners may be resolved in the context of type-(i) solutions at best TP rates of ~ 70%—far above the same estimates in type-(ii) solutions.

Type-(i) and -(ii) solutions are found to be functionally distinct from each other, with the former presenting a larger near-native content of mutual information correctly distributed among amino acid contacts. Particularly important, that finding supports the notion that their differentiation based on TP rates is not just a theoretical construct but instead has a biological meaning associated with how much functional information is recovered and how accurately distributed this information is. Type-(i) solutions may therefore correspond to reliable results for predictive purposes^1^, more likely obtained via $\hat{I}_{AB}$ maximization across protein systems with a minimum critical number of amino acid contacts on their interaction surfaces (N > 200).

Finally, as a special case of a highly specific system of paralogs, HK-RR interactions are resolved here at very low TP rates following $\hat{I}_{AB}$ maximization, which is consistent with TP rates reported in the literature^19^ employing other more complex optimization algorithms, such as DCA-IPA^13^. As shown in Fig. 6, the HK-RR system was found not to present type-(i) degeneracy and, as such, its TP rates sharply deteriorate with M $\ge \hspace{0.17em}$32 sequences per genome and cannot be improved by any means. Exclusive existence of type-(ii) errors in the HK-RR system thus suggests another layer of complexity that sequence diversity and specificity may add to the problem. Investigation of these aspects as key determinants for error sources (i) and (ii) is therefore another important perspective of the presented work. In this direction, we speculate that HK-RR pairs within the same genome are highly specific and this is the reason why there is no type (i) error in this system. In contrast, systems with only one pair of interacting proteins per genome do not suffer selective pressure to avoid cross-binding homologs occurring in other species and, therefore, present both type (i) and type (ii) errors.

Overall, the investigations performed in this work provide some clarifications into the general problem of protein coevolution from the perspective of sequence diversity. It is difficult to say to which point homologous sequences were selected to selectively bind to their native partners since there is a huge degeneracy in the space of possible sets of partners. Despite the intrinsic complexity of the problem of specific protein partners prediction for large sequence ensembles, the novel theoretical insights presented here might provide relevant information for future studies and should contribute to advancing our knowledge in the field.

## Methods

**Figure 7:**
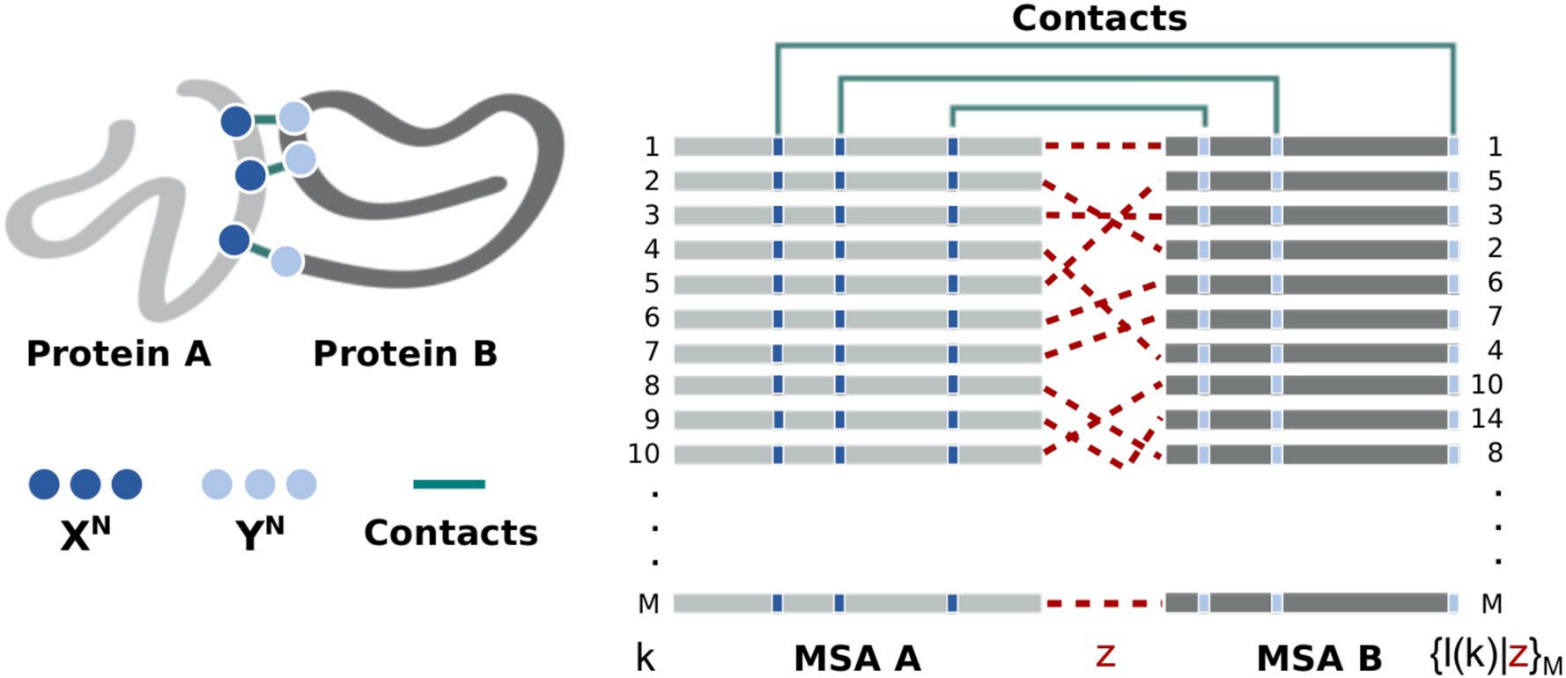
Structural contacts mapped into *M*-long multi-sequence alignment of protein interologs *A* and *B*. A set of pairwise protein–protein interactions is defined by associating each sequence *l* in MSA *B* to a sequence *k* in MSA *A* in one unique arrangement, $\{ l(k){\mid }z\}$, determined by the coevolution process $z$ to which these protein families were subjected. This figure was created with Inkscape (https://inkscape.org/).

Consider two interacting protein families, *A* and *B*. It is possible to construct two MSAs, MSA *A* and MSA *B*, containing *M* sequences from families *A* and *B*, respectively. A specific coevolution process $z \in \{ 1, \ldots ,M!\}$ associates each sequence *l* in MSA *B* to a sequence *k* in MSA *A* in a unique arrangement of size *M* (see Fig. 7). Given that members of *A* and *B* interact via formation of *N* independent amino acid contacts at molecular level, it is possible to extract from these MSAs only the columns corresponding to sites that are in contact, belonging to the complex interface. In this context, the interacting amino acids of families A and B are described by two $N$-length blocks of discrete stochastic variables, $X^{N} = (X_{1} , \ldots ,X_{N} )$ and $Y^{N} = (Y_{1} , \ldots ,Y_{N} )$, with associated probability mass functions (PMFs) $\{ \rho (x_{1} \ldots x_{N} ),\;\rho (y_{1} \ldots y_{N} ),\;\rho (x_{1} \ldots x_{N} ,\;y_{1} \ldots y_{N} {\mid }z){\mid }x_{i} ,\;y_{i} \in \Omega ,\;\;\forall i \in \{ 1, \ldots ,N\} \}$. Here, the alphabet $\Omega$ has size 21 and contains all 20 amino acids and the gap symbol '–'. Note that only the joint PMF will depend on process *z*.

Here, we approximate each site-specific PMF $\{ \rho (x_{i} ),\;\rho (y_{i} ),\;\rho (x_{i} ,\;y_{i} {\mid }z){\mid }i \in \{ 1, \ldots ,N\} \}$ by the empirical amino acid frequencies $\{ f(x_{i} ),\;f(y_{i} ),\;f(x_{i} ,\;y_{i} {\mid }z){\mid }i \in \{ 1, \ldots ,N\} \}$ obtained from the concatenated MSAs. Note that each coevolution process *z* determines a specific concatenation, as illustrated in Fig. 7. It means that, essentially, the search will be guided by the amount of information *X*^*N*^ stored about *Y*^*N*^ conditional to different coevolution processes *z*.

### Shannon mutual information

The Shannon mutual information contained on the interface of interacting proteins A and B conditional to a given coevolution process z is calculated as follows

1 $$\begin{aligned} \hat{I}_{AB} & = \frac{1}{N}I(X^{N} ;\;Y^{N} {\mid }z) = \frac{1}{N}\sum\limits_{i = 1}^{N} {} I(X_{i} ;\;Y_{i} {\mid }z) \\ & = \frac{1}{N}\mathop \sum \limits_{\Omega x\Omega } f(x_{i} ,\;y_{i} {\mid }z)\ln \left( {\frac{{f(x_{i} ,\;y_{i} {\mid }z)}}{{f(x_{i} )f(y_{i} )}}} \right),\quad x_{i} ,\;y_{i} \in \Omega \\ \end{aligned}$$

where *N* is the number of contacts at the *AB* complex interface, $f(x_{i} )$ is the empirical frequency of $x_{i}$ as a realization of $X_{i}$, $f(y_{i} )$ is the empirical frequency of $y_{i}$ as a realization of $Y_{i}$, and $f(x_{i} ,\;y_{i} {\mid }z)$ is the empirical frequency of pair $(x_{i} ,\;y_{i} )$ as a realization for the i-th contact given a specific coevolution process *z*.

The empirical values of single and joint frequencies were corrected considering a pseudocount, as follows

$$f_{i} (x_{i} ) \leftarrow (1 - \lambda )f_{i} (x_{i} ) + \frac{\lambda }{Q}$$

$$f_{ij} (x_{i} ,\;x_{j} {\mid }z) \leftarrow (1 - \lambda )f_{ij} (x_{i} ,\;x_{j} {\mid }z) + \frac{\lambda }{{Q^{2} }}$$

where, *Q* is the size of alphabet $\Omega$ and $\lambda$ is the pseudocount parameter. In this work, we adopt a small pseudocount of $\lambda = 0.001$.

The joint entropy of the interface was calculated for individual contacts

$$H(X_{i} ,\;Y_{i} {\mid }z) = f(x_{i} ,\;y_{i} {\mid }z)\ln (f(x_{i} ,\;y_{i} {\mid }z))$$

where $f(x_{i} ,\;y_{i} {\mid }z)$ is the empirical frequency of pair $(x_{i} ,\;y_{i} )$ as a realization for the i-th contact given a specific coevolution process *z*. Afterwards, the regularization $I_{AB} /H_{AB}$ was obtained according to

$$I_{AB} /H_{AB} = \mathop \sum \limits_{i = 1}^{N} I(X_{i} ;\;Y_{i} {\mid }z)/H(X_{i} ,\;Y_{i} {\mid }z)$$

where N is the number of contacts.

### Systems under investigation

Protein complexes under investigation are shown in Table S1. MSAs *A* and *B* for all protein families were obtained from Ovchinnikov and coworkers^25^. Amino acid contacts defining the discrete stochastic variables *X*^*N*^ and *Y*^*N*^ were identified from the x-ray crystal structure of the bound state of a representative protein pair from families *A* and *B* using a typical contact definition considering maximum separation distance of 8 Å between amino acids carbon beta. The full dataset of protein systems validated in^25^ was considered here, except for systems 2Y69_BC, 2ONK_AC, 3A0R_AB, 3RPF_AC, and 4HR7_AB, which were considered outliers in terms of M/N values 469.3, 87.7, 192.3, 150.6, and 45.3 significantly larger than their typical estimates described in Table S1.

Additionally, the HK-RR standard dataset containing around 5000 sequences, coming from around 450 bacterial genomes from the P2CS database^22–24^ was included. This paired MSA was produced and validated by Bitbol and coworkers^13^ in paralog matching experiments. The PDB complex 5UHT (chains A and B) was selected as a representative for this system. The reason for including this system containing paralogous proteins is to have a baseline for comparison with previous related studies.

### Genetic algorithm

The mutual information contained on the interface of the protein complexes, calculated as described in Eq. (1), was maximized using a Genetic Algorithm (GA, Algorithm S1). For each of the protein complexes considered, six independent optimization trajectories were obtained, starting from different randomly generated populations. Each optimization was performed with a population of eight individuals with unique genomes encoding a specific concatenation *z* of MSAs A and B. In each generation, the elite (top-50% individuals with the best fitness) reproduces and replaces the remaining 50% individuals with lower fitness with new individuals with genomes that are mutated copies of the elite. A mutation in the genome of an individual consists of swapping positions of two sequences on MSA *B*, and thereby slightly changing the concatenation *z*. The fitness of the individuals is calculated in each generation and corresponds to the total interface mutual information obtained considering an individual unique genome, i.e., a specific concatenation of MSAs A and B. The optimization was stopped after a predefined number of 50,000 generations was reached.

**Figure 8:**
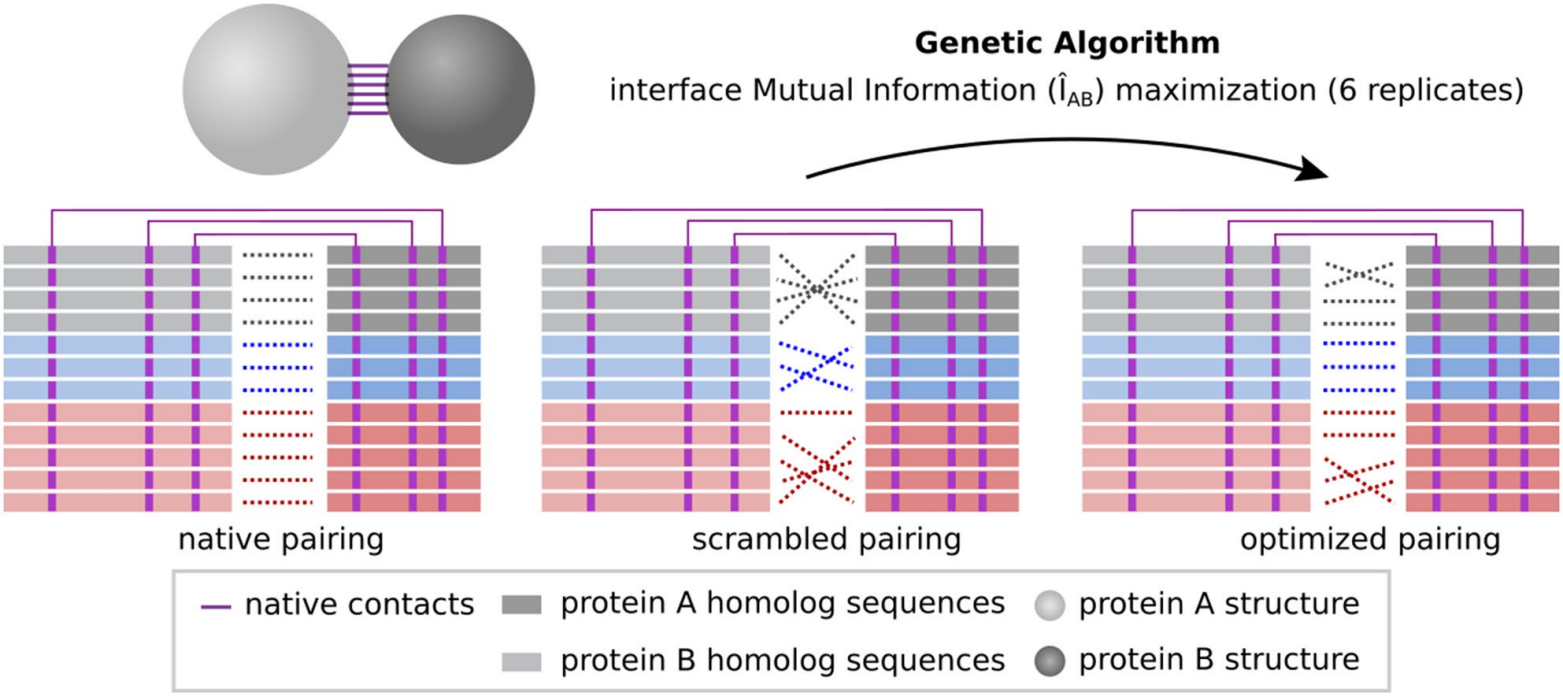
Scheme showing interface mutual information ($\hat{I}_{AB}$) optimization process for the HK-RR standard dataset. It starts from a within-species scrambled MSA concatenation and reaches an optimized concatenation. Different species are shown in different colors. Only physically coupled MSA position pairs (shown in purple) are taken into account and only within-species changes are made in each generation. This figure was created with Inkscape (https://inkscape.org/).

A slightly different optimization procedure was implemented for the special case of the HK-RR standard dataset (Fig. 8). In this case, the initial population is composed of within-species scrambled solutions and, in each generation, only within-species changes are allowed. More specifically, each time a new mutated individual is generated, one of the species that compose the MSA is randomly selected, and a change in the concatenation within this species is performed. The optimization was stopped after a predefined number of 100,000 generations was reached.

The optimal set of parameters for the GA were derived from a series of tests performed on six representative systems. In each test, one of these parameters varied, assuming a range of values while all other parameters remained fixed (Table S2). All tests were performed with a predefined seed for the random number generator, which means that the starting point and the sequence of mutations performed are constant for all trajectories of the same system. This was done to ensure that any effects observed in the final results were due solely to variations in the GA parameters.

Figure S8 shows how parameter values correlated with relative $\hat{I}_{AB}$ at the end of test trajectories. Given that both the number of individuals and the elite proportion correlated positively with relative $\hat{I}_{AB}$ (Figure S8A,B), the values selected for these parameters were the maximum tested, i.e., 8 and 0.5, respectively. The number of mutations, on the other hand, correlated negatively with relative $\hat{I}_{AB}$ (Figure S8C), thus the value selected for this parameter was 1. Results for parameter $\lambda$ were not so conclusive (Figure S8D) and, since this parameter was set to 0.001 in previous work^15^, its value was maintained the same. As shown in Figure S9, GA parameters do not influence TP rates observed at the end of trajectories thus supporting that our conclusions are robust over GA parameters, with the possible exception of $\lambda$, which will be investigated in future work.

### Assessment of optimized solutions accuracy

**Figure 9:**
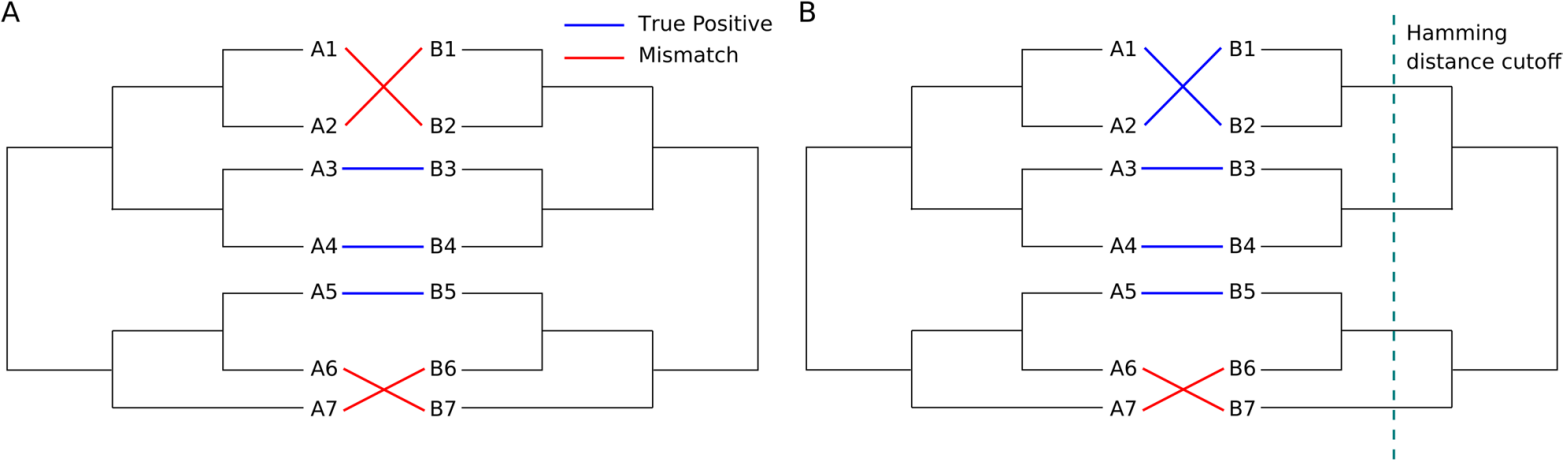
Mismatch discounting based on a Hamming distance cutoff. Scheme showing how the accuracy of the same MSA concatenation would be assessed with (**B**) and without (**A**) mismatch discounting. This figure was created with Inkscape (https://inkscape.org/).

The true positive (TP) rates of optimized concatenations obtained at the end of the genetic algorithm (GA) $\hat{I}_{AB}$ maximization trajectories were calculated in two different manners: with and without mismatch discounting. TP rate assessment without mismatch discounting consists simply of counting how many sequence partners were correctly paired in the target solution and divided by the total number of sequences (Fig. 9A). TP rate assessment with mismatch discounting, on the other hand, consists of counting how many sequences were paired either with their correct partner or with a partner that is close enough to the correct one in terms of Hamming distance (Fig. 9B). Hence, mismatch discounting depends on a predefined Hamming distance cutoff, below which sequences are considered similar enough for the mistakes to be forgiven. Here, we consider the 20th percentile of a given protein family B distance distribution as the predefined cutoff for mismatch discounting. Figure S1 shows that the relaxation of that parameter does not affect qualitatively the results.

A K-Nearest Neighbors (KNN) classifier was used to investigate if MSA pairing solutions with trivial and non-trivial error sources scattered differently in the space of relative $\hat{I}_{AB}$ against correlation of individual MI values with the native solution, $r(\hat{I}(X_{i} ;\;Y_{i} ),\;\hat{I}_{nat}^{T} (X_{i} ;\;Y_{i} ))$. All type-(i) and type-(ii) solutions obtained were used to train a KNN classifier with default scikit-learn (https://scikit-learn.org) parameters, except for the number of neighbors (K). Values of K were tested ranging from 2 to 20, but little variation in the accuracy score was observed, with scores ranging from 0.76 to 0.87. Therefore a value of K = 10 was chosen as a compromise between a possible overfit when considering too few neighbors and losing accuracy when considering too many neighbors (results for other values of K are shown in Figure S2). The accuracy score was calculated using the scikit-learn function *.score*() on the model inferred by the KNN classifier. This function indicates how well the model fits the provided data points, i.e., it calculates the accuracy on the training set.

## Supplementary Information

Supplementary Information.
