# Supplementary material for: Trivial and nontrivial error sources account for misidentification of protein partners in mutual information approaches
Source: Sci Rep. 2021 Mar 25;11:6902. doi: 10.1038/s41598-021-86455-0 (PMC7994710; doi:10.1038/s41598-021-86455-0)
Supplement: Supplementary file 1 — Supplementary Information. [file 41598_2021_86455_MOESM1_ESM.pdf]

Trivial and nontrivial error sources account for misidentification of protein partners in mutual information approaches

Supporting Information

Camila Pontes‡[1], Miguel Andrade‡[1], José Fiorote[1] and Werner Treptow*[1]

‡ these authors contributed equally
* corresponding author

Authors Affiliation**:**
[1]Laboratório de Biologia Teórica e Computacional (LBTC), Universidade de Brasília DF, Brasil

Supporting Information Placeholder

Corresponding Author:
Werner Treptow
Laboratório de Biologia Teórica e Computacional (LBTC), Universidade de Brasília DF, Brazil
+55 61 3107-3098
treptow@unb.br

Keywords:
Coevolution; Mutual Information; Protein-Protein Interaction

Author contributions. CP, MA and WT designed research; CP, MA and JF performed research; CP, MA, JF and WT analyzed data; CP and WT wrote the original and the reviewed manuscript; CP and MA contributed equally to this work.

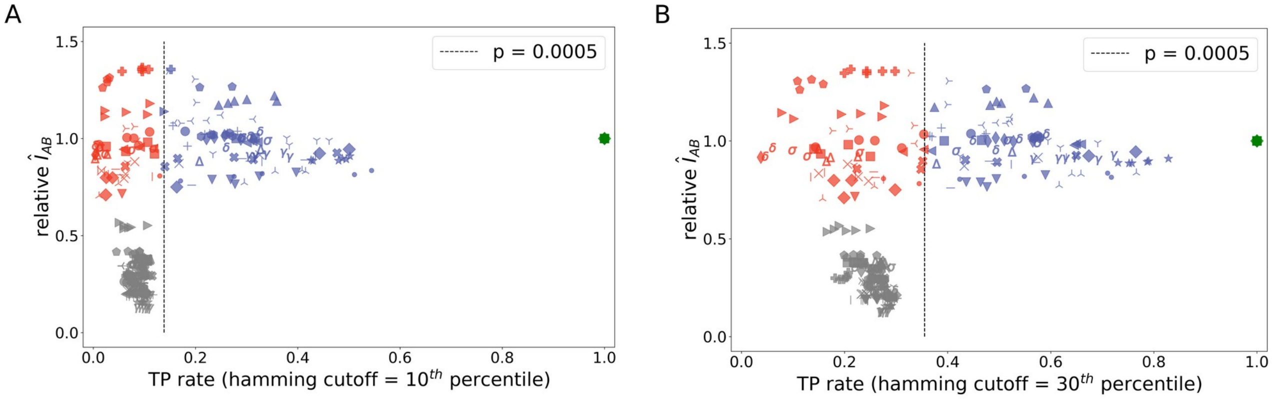

Figure S1. TP rate of random, optimized and native MSA concatenations discounting wrong pairings among sequences within low Hamming distance, within the 10th (A) and 30th (B) percentiles of the distance distribution. Optimized solutions with TP rate greater than 14% (A) and 36% (B) (p-value = 0.0005) at the chosen threshold are shown in blue, while optimized solutions with TP rate lower than 14% (A) and 36% (B) at the chosen threshold are shown in red. Random solutions are shown in gray. This figure was generated using matplotlib v3.1.2 (https://matplotlib.org).

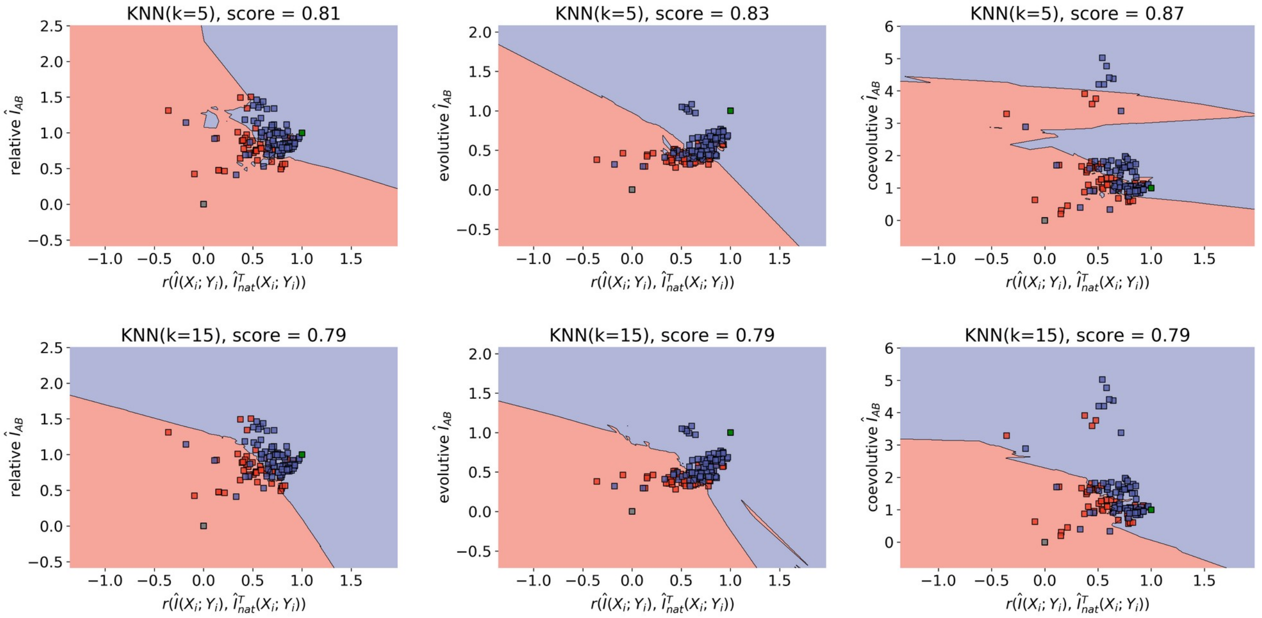

Figure S2. Optimized concatenation solutions scattered across the space of relative interface mutual information (MI), $\hat{I}_{AB}$, against Pearson correlation between optimized and native MI vectors, $r\left(\hat{I}\left(X_i;Y_i\right),\hat{I}^T_{nat}\left(X_i;Y_i\right)\right)$. Type-*(i)* solutions are shown in red and type-*(ii)* solutions are shown in blue. The bidimensional space was separated by a k-nearest neighbors (KNN) classification algorithm (default Python 3 scikit-learn implementation, k = 5 or 15). Native and scrambled concatenations were plotted afterwards in the same space and are shown in green and gray, respectively. Analogous plots were generated for the evolutive and coevolutive components of $\hat{I}_{AB}$. The decomposition was performed according to [15]. This figure was generated using sci-kit learn v0.22.2 (https://scikit-learn.org) and mlxtend v0.18.0 (http://rasbt.github.io/mlxtend/).

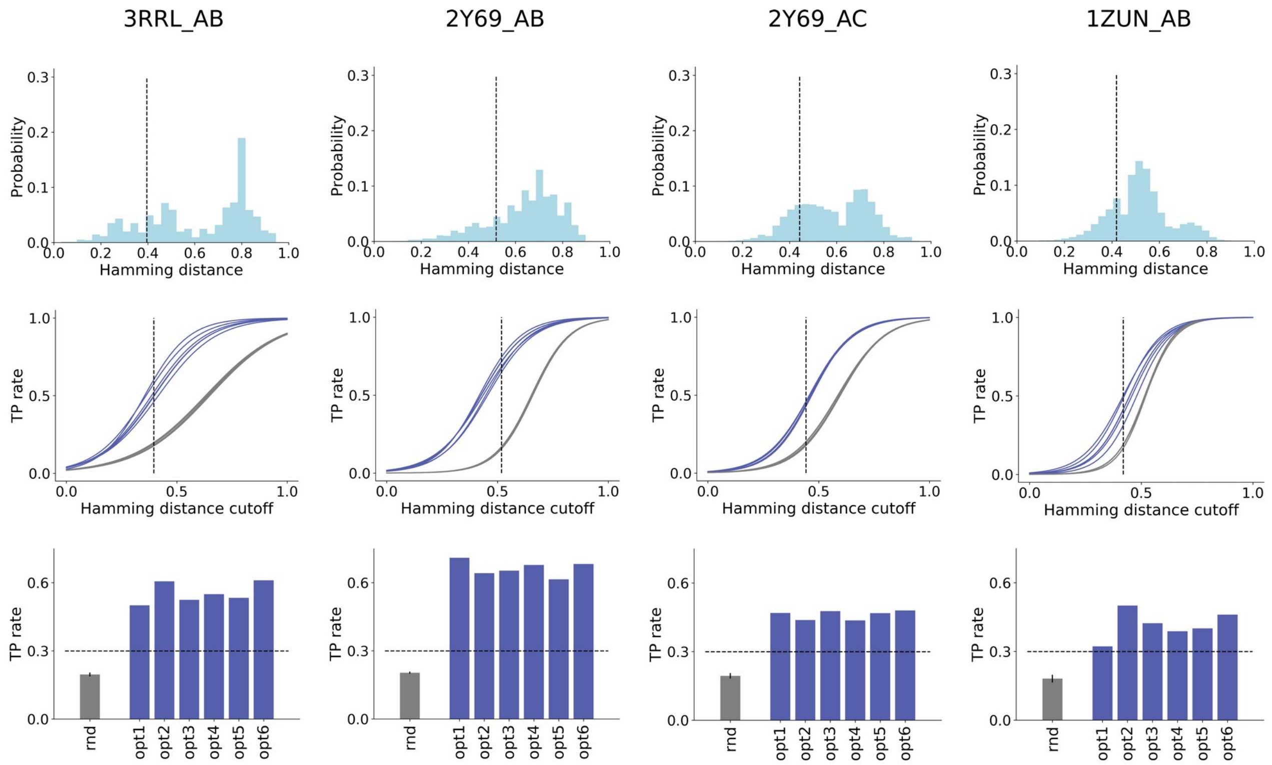

Figure S3. Group 1 – all solutions are type-*(i)*, blue. (1st) Hamming distance distribution of MSA B. (2nd) True positive (TP) rate for different Hamming distance discounts. The 20th percentile is shown with a dashed line, random solutions in gray, optimized solution in blue. (3rd) TP rates of random (rnd) and optimized (opt1-6) solutions at 20th percentile Hamming distance cutoff. The significance value is shown with a dashed line (p=0.0005). This figure was generated using matplotlib v3.1.2 (https://matplotlib.org).

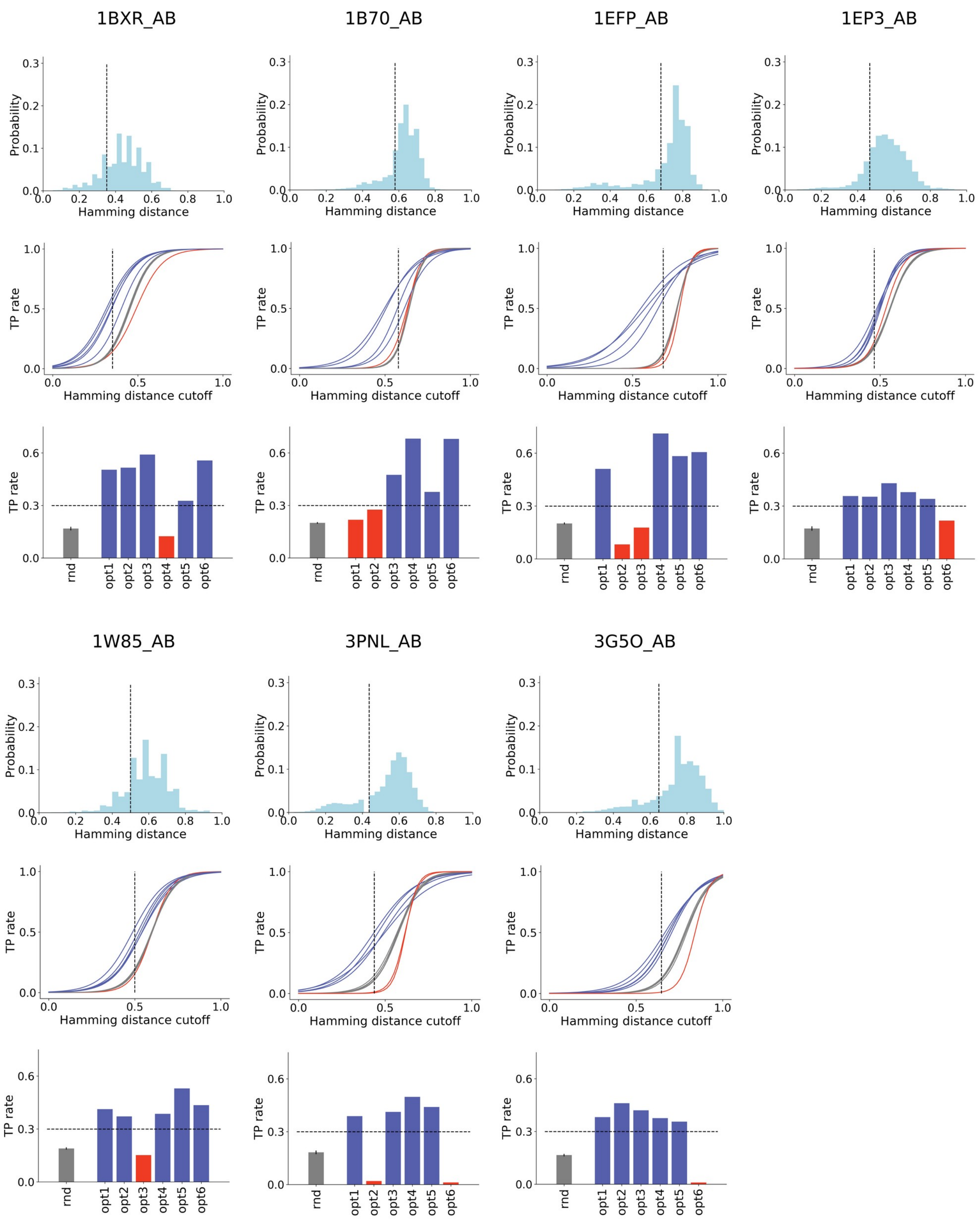

Figure S4. Group 2 – majority of type-*(i)* solutions, blue. (1st) Hamming distance distribution of MSA B. (2nd) True positive (TP) rate for different Hamming distance discounts. The 20th percentile is shown with a dashed line, random solutions in gray, optimized solution in blue. (3rd) TP rates of random (rnd) and optimized (opt1-6) solutions at 20th percentile Hamming distance cutoff. The significance value is shown with a dashed line (p=0.0005). This figure was generated using matplotlib v3.1.2 (https://matplotlib.org).

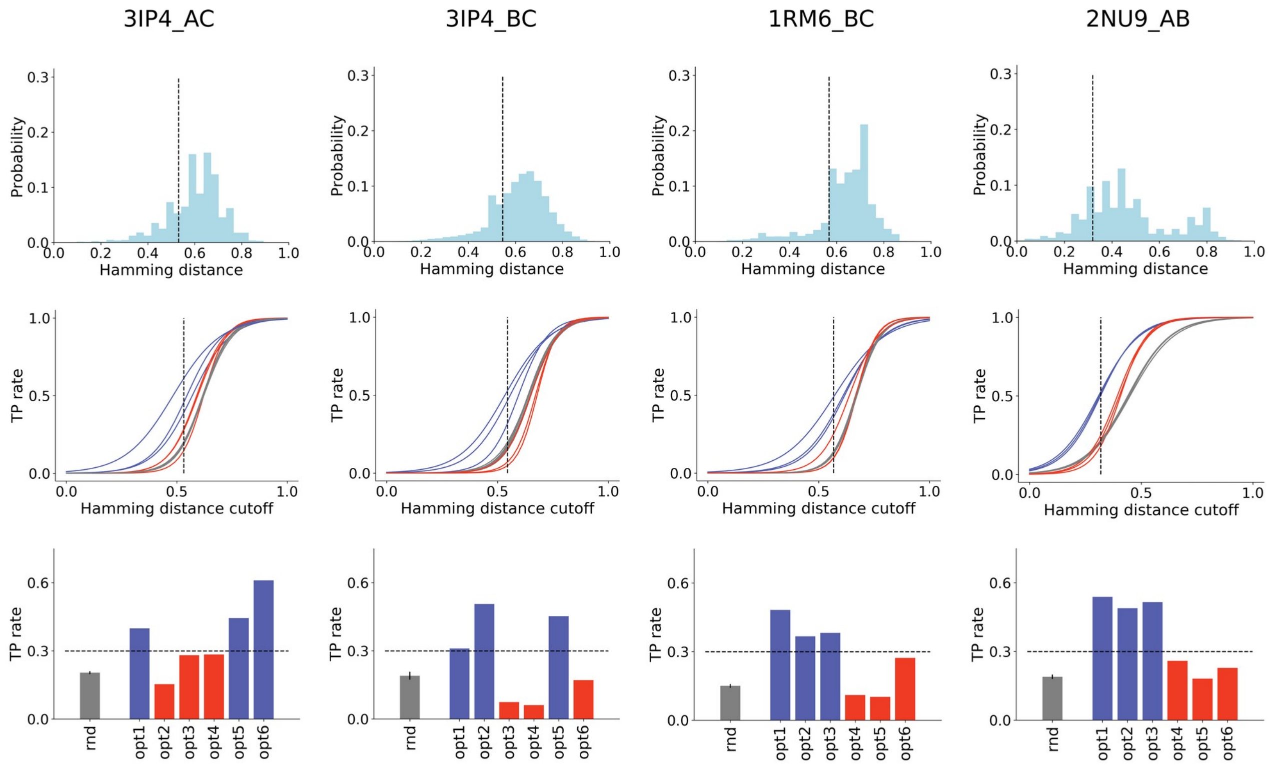

Figure S5. Group 3 – mixed type-*(i)* blue and type-*(ii)* red solutions. (1st) Hamming distance distribution of MSA B. (2nd) True positive (TP) rate for different Hamming distance discounts. The 20th percentile is shown with a dashed line, random solutions in gray, optimized solution in blue. (3rd) TP rates of random (rnd) and optimized (opt1-6) solutions at 20th percentile Hamming distance cutoff. The significance value is shown with a dashed line (p=0.0005). This figure was generated using matplotlib v3.1.2 (https://matplotlib.org).

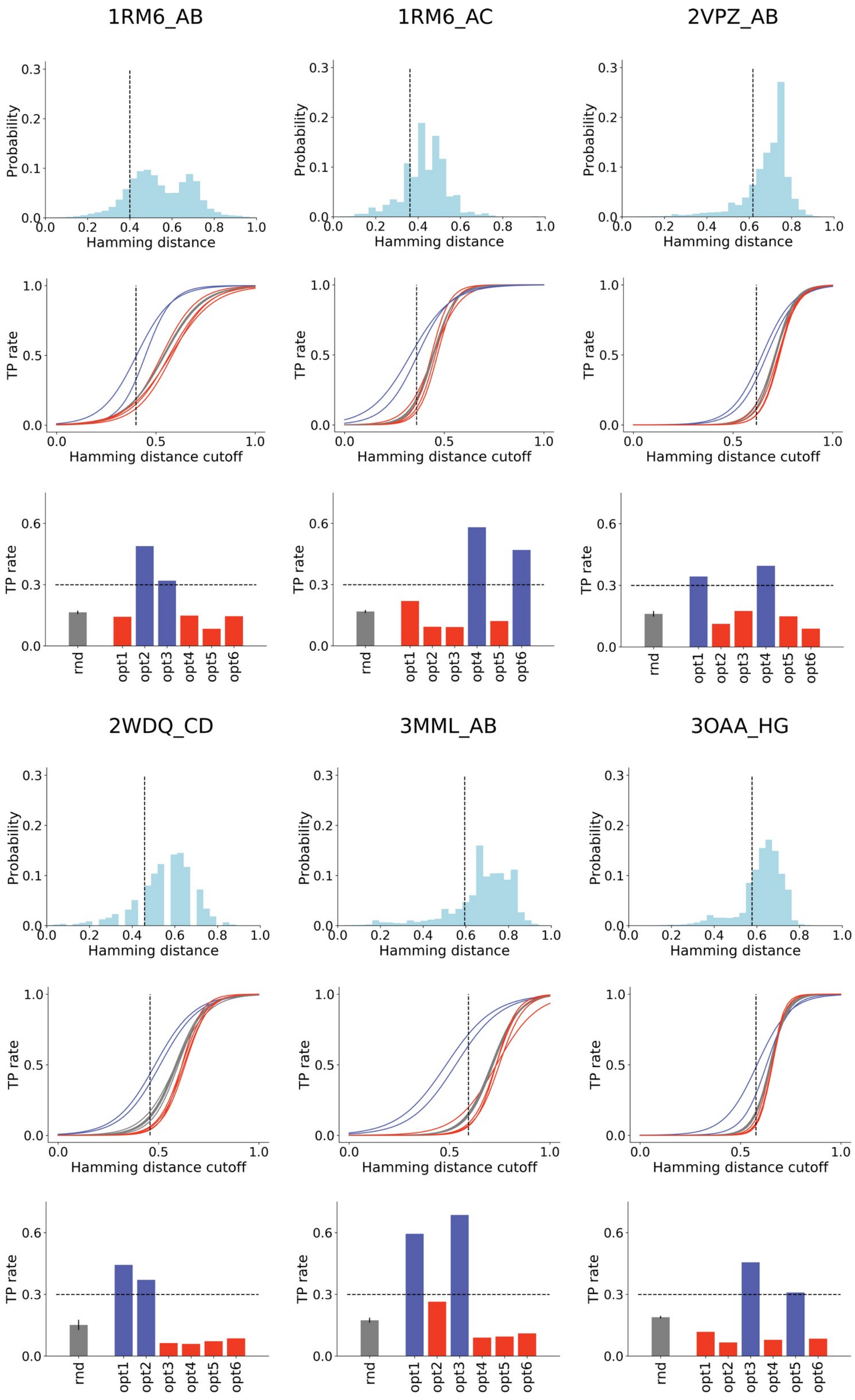

Figure S6. Group 4 – majority of type-*(ii)* solutions, red. (1st) Hamming distance distribution of MSA B. (2nd) True positive (TP) rate for different Hamming distance discounts. The 20th percentile is shown with a dashed line, random solutions in gray, optimized solution in blue. (3rd) TP rates of random (rnd) and optimized (opt1-6) solutions at 20th percentile Hamming distance cutoff. The significance value is shown with a dashed line (p=0.0005). This figure was generated using matplotlib v3.1.2 (https://matplotlib.org).

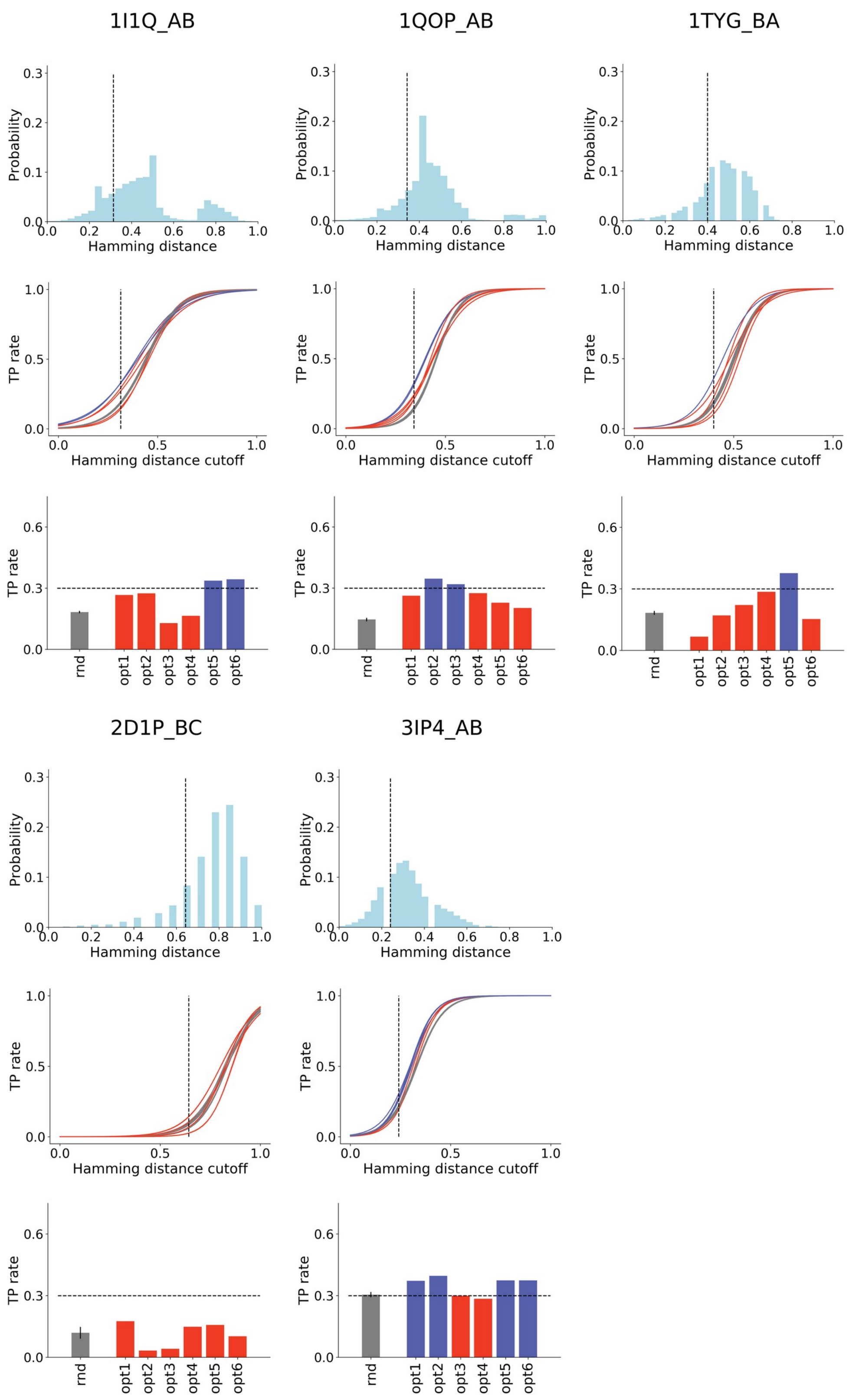

Figure S7. Group 5 – optimized solutions non-distinguishable from random. (1st) Hamming distance distribution of MSA B. (2nd) True positive (TP) rate for different Hamming distance discounts. The 20th percentile is shown with a dashed line, random solutions in gray, optimized solution in blue. (3rd) TP rates of random (rnd) and optimized (opt1-6) solutions at 20th percentile Hamming distance cutoff. The significance value is shown with a dashed line (p=0.0005). This figure was generated using matplotlib v3.1.2 (https://matplotlib.org).

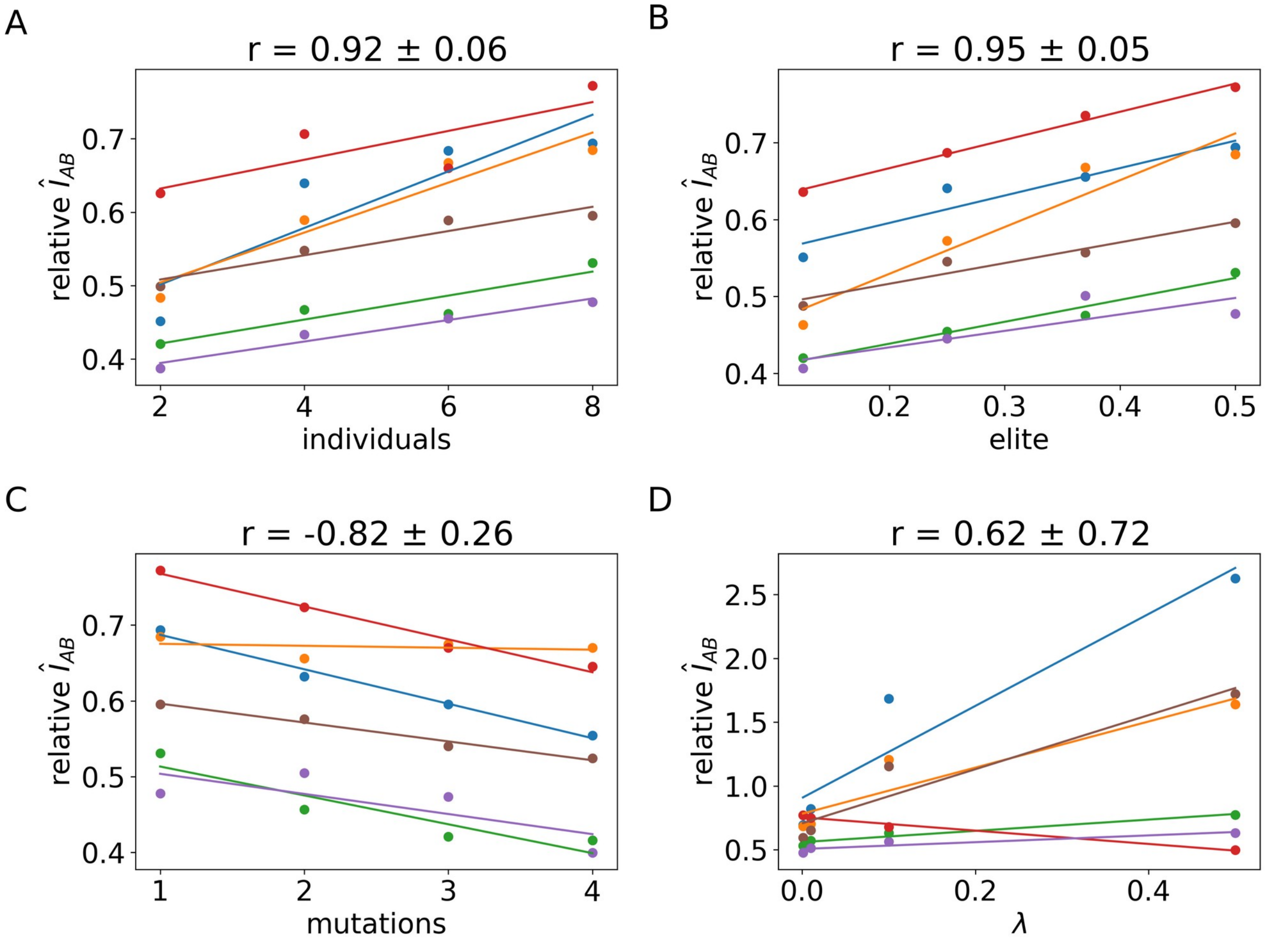

Figure S8. Analysis of relative $\hat{I}_{AB}$ values reached at the end of genetic algorithm parameters test trajectories considering six representative systems: 1BXR_AB, 3MML_AB, 2NU9_AB, 1RM6_AB, 3IP4_AC and 3G5O_AB. The parameters tested were: population size (A), elite (B), number of mutations (C), and pseudocount parameter (D). While one parameter was tested, the others were fixed in the following default values: 8, 0.5, 1 and 0.001, respectively. All trajectories ended after 5,000 generations. The average Pearson correlation is shown on top of each plot considering all systems (n = 6). This figure was generated using matplotlib v3.1.2 (https://matplotlib.org).

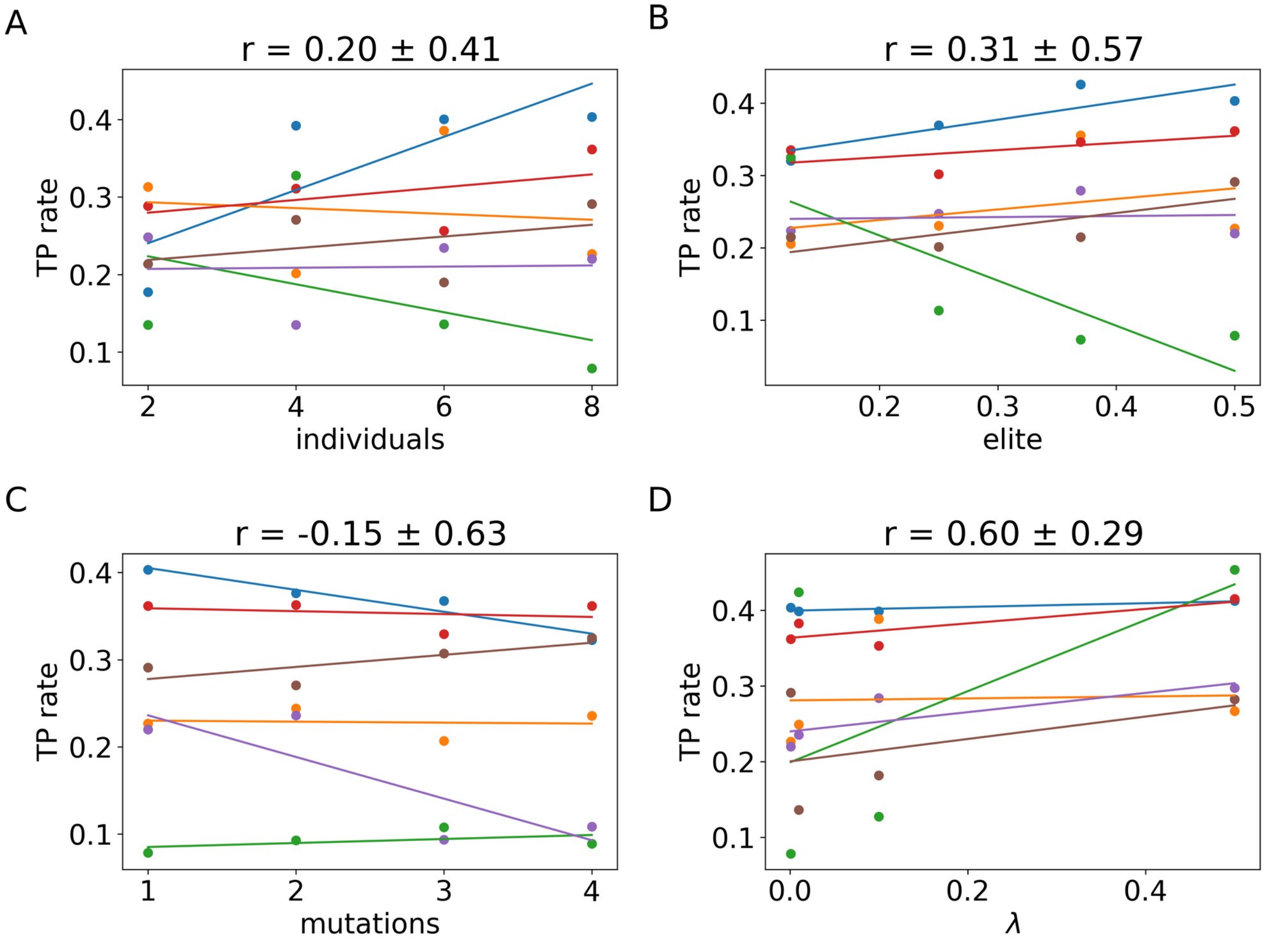

Figure S9. Analysis of true positive (TP) rates on genetic algorithm parameters test trajectories considering six representative systems: 1BXR_AB, 3MML_AB, 2NU9_AB, 1RM6_AB, 3IP4_AC and 3G5O_AB. The parameters tested were: population size (A), elite (B), number of mutations (C), and pseudocount parameter (D). While one parameter was tested, the others were fixed in the following default values: 8, 0.5, 1 and 0.001, respectively. All trajectories ended after 5,000 generations. The average Pearson correlation is shown on top of each plot considering all systems (n = 6). This figure was generated using matplotlib v3.1.2 (https://matplotlib.org).

Table S1. Protein complexes considered in the study. M is the number of sequences in the multi-sequence alignment and N is the number of contacts in the interface, following an 8Å cutoff definition.

#	Description	PDB ID	Chains	M	N	M/N
1	Dihydroorotate dehydrogenase B (4-mer)	1EP3	A, B	552	91	6.1
2	Thiazole synthase/ThiS (8-mer)	1TYG	A, B	746	80	9.3
3	Carbamoyl phosphate synthetase (8-mer)	1BXR	A, B	1,004	154	6.5
4	3-oxoadipate coA-transferase (4-mer)	3RRL	A, B	1,330	161	8.3
5	Toxin-antitoxin complex RelBE2 (4-mer)	3G5O	A, B	904	92	9.8
6	Bovine cytochrome C oxidase (13-mer)	2Y69	A, B	1,484	246	6.0
7	Bovine cytochrome C oxidase (13-mer)	2Y69	A, C	863	210	4.1
8	Phenylalanyl-tRNA synthetase (4-mer)	1B70	A, B	1,108	255	4.3
9	Electron transfer flavoprotein (2-mer)	1EFP	A, B	1,347	229	5.9
10	Anthranilate synthase (4-mer)	1I1Q	A, B	1,204	91	13.2
11	Tryptophan synthase (4-mer)	1QOP	A, B	1,155	102	11.3
12	4-hydroxybenzoyl-CoA reductase (6-mer)	1RM6	A, B	1,604	71	22.6
13	4-hydroxybenzoyl-CoA reductase (6-mer)	1RM6	A, C	1,534	154	10.0
14	4-hydroxybenzoyl-CoA reductase (6-mer)	1RM6	B, C	1,481	93	15.9
15	Pyruvate dehydrogenase E1 (5-mer)	1W85	A, B	1,537	121	12.7
16	GTP-Regulated ATP Sulfurylase (2-mer)	1ZUN	A, B	649	140	4.6
17	TusBCD proteins (6-mer)	2D1P	B, C	216	40	5.4
18	Succinyl-CoA Synthetase (4-mer)	2NU9	A, B	798	144	5.5
19	Polysulfide reductase (6-mer)	2VPZ	A, B	676	119	5.7
20	Succinate:quinone oxidoreductase (4-mer)	2WDQ	C, D	221	43	5.1
21	GatCAB (3-mer)	3IP4	A, B	782	94	8.3
22	GatCAB (3-mer)	3IP4	A, C	879	146	6.0
23	GatCAB (3-mer)	3IP4	B, C	689	122	5.6
24	Allophanate Hydrolase (4-mer)	3MML	A, B	1,067	116	9.2
25	F1-ATP synthase (8-mer)	3OAA	H, G	886	179	4.9
26	DhaK-DhaL (4-mer)	3PNL	A, B	902	113	8.0
27	*Thermotoga maritima* HK853-RR468 (4-mer)	5UHT	A, B	5,110	33	

Table S2. Genetic algorithm parameters values tested on representative systems 1BXR_AB, 3MML_AB, 2NU9_AB, 1RM6_AB, 3IP4_AC and 3G5O_AB. In each test, one of these parameters varied to assume all its possible values while all other parameters remained fix in the reference value (*).

Population size	Elite (% of population)	Number of mutations	Pseudocount
2	12.5% (1/8)	**1***	**0.001***
4	25% (2/8)	2	0.01
6	37.5% (3/8)	3	0.1
**8***	**50% (4/8)***	4	0.5

Algorithm S1. Simplified Python implementation of the genetic algorithm used for mutual information optimization.

```
# User defined parameters
GENERATIONS = 50000
MUTATIONS = 1
INDIVIDUALS = 8
ELITE = 4
LAMBDA = 0.001

# Read and encode MSA
msa_a = readMSA("msa_a.fasta")
msa_b = readMSA("msa_b.fasta")

# Read and map contacts to MSA
contacts = readContacts("contacts_8A.txt")
col_pairs = mapContactsToMSAs(contacts, msa_a, msa_b)

# Fitness function
def fitness(genome):
	# Concatenates MSA A and B and extract relevant column pairs
	coev_model = getCoevolutionModel(genome, msa_a, msa_b, col_pairs)
	# Calculate single-site amino acid frequencies
	site_freqs = calculateSiteFreqs(coev_model, LAMBDA)
	# Calculate double-site amino acid frequencies
	pair_freqs = calculatePairFreqs(coev_model, LAMBDA)
	# Calculate Shannon's mutual information matrix
	mi = calculateMI(site_freqs, pair_freqs)

	return sum(mi)

# Prepare initial population
population = []
for n in range(INDIVIDUALS):
	# Generate random concatenation for MSA A and B
	new_genome = generateRandomGenome(len(msa_a))
	population.append([new_genome, fitness(new_genome)])
population.sort(key = lambda x : x[1], reverse = True)

# Run optimization
for g in range(GENERATIONS):
	for i in range(ELITE):
		genome = population[i][0]
		for j in range(MUTATIONS):
			# Swap two indexes in genome
			mutate(genome)
		# Replace low fitness individuals
		population[INDIVIDUALS – ELITE + i] = [genome, fitness(genome)]
	population.sort(key = lambda x : x[1], reverse = True)
```
